# Nutritional Status and Serum Levels of Micronutrients in an Elderly Group Who Participate in the Program for Complementary Food in Older People (PACAM) from the Metropolitan Region, Santiago de Chile

**DOI:** 10.3390/nu14010003

**Published:** 2021-12-21

**Authors:** Migdalia Caridad Arazo-Rusindo, Rommy N. Zúñiga, Pablo Cortés-Segovia, Sergio Benavides-Valenzuela, Francisco Pérez-Bravo, Oscar Castillo-Valenzuela, María Salomé Mariotti-Celis

**Affiliations:** 1 Department of Biotechnology, Universidad Tecnológica Metropolitana, Las Palmeras 3360, Ñuñoa, Santiago 7800003, Chile; migdalia.arazo@utem.cl (M.C.A.-R.); rommy.zuniga@utem.cl (R.N.Z.); 2 Faculty of Medicine, Nutrition and Dietetics School, Universidad Finis Terrae, Pedro de Valdivia 1509, Providencia, Santiago 7501015, Chile; pcortes@uft.cl; 3 Núcleo de Investigación en Agroalimentos y Nutrición Aplicada, Universidad Adventista de Chile, Camino a las Mariposas s/n, Chillan 3780000, Chile; sergiobenavides@unach.cl; 4 Institute of Nutrition and Food Technology (INTA), University of Chile, Santiago 7830489, Chile; 5 Nutrigenomics Laboratory, Nutrition Department, Faculty of Medicine, University of Chile, Santiago 7830489, Chile

**Keywords:** older adults, nutritional status, micronutrients intake, serum micronutrients levels, fortified foods, PACAM

## Abstract

The increase in the Chilean elderly population has promoted public policies to favor an adequate nutrition in later life. This study evaluated the nutritional status, micronutrients intake and serum micronutrients levels of an elderly group beneficiary of the PACAM from the Metropolitan Region, Santiago de Chile. Anthropometric and dietary survey (24 h food recalls) were assessed in 182 elderly individuals (60 and 80 years old). Blood serum collection was used to measure the micronutrient status. The sample was comprised by 12.6%, 46.1%, 28.0% and 13.2% of underweight, normal weight, overweight and obese subjects, respectively. Women presented 11% of underweight, 45% of normal weight and 44% of overweight and obese, while men—18%, 50% and 32%, respectively. Only the 63% of the elderlies consumed PACAM foods, reaching average daily intakes below (50%) the recommended daily serving. Serum deficiencies of 25-hydroxyvitamin D (88%), vitamin B_12_ (33%) and calcium (36%) were observed, being the highest ones in the PACAM foods women (60–75 years old). Chilean elderlies presented mainly a normal weight; however, an important proportion of overweight/obese subjects was observed. Although PACAM foods consumption significantly increased the micronutrient intake, it was not enough to ensure an adequate serum micronutrient levels in the elderly.

## 1. Introduction

The World Health Organization (WHO, Geneva, Switzerland) defines aging as a gradual and irreversible physiological process that involves changes in tissues and body functions over time [1]. Aging is a process that goes on over the entire adult life span of any living thing, and it takes place in a cell, an organ, or the total organism with the passage of time. These changes cause limitations in the adaptability of the organism in relation to its environment. Thus, the aging population will increasingly need special attention to improve their quality of life [2].

Globally, life expectancy at birth has dramatically increased due to the declining of infant and child mortality rates, combined with an improved access to health care. As a result, the world is experiencing a process of rapid population aging. By 2050, the global population aged 65 years or over is expected to be more than quadruple that in 2004 (461 million), reaching nearly 2.1 billion [3]. The aging of the world population calls for important challenges to countries and health systems. A response to these challenges has been implementing programmes and policies targeted to older people, under the premise that they require customized solutions to their needs [1].

Chile is not the exception, being among the countries with fastest growth of life expectancy (82.1 and 77.3 years old for women and men, respectively) [4]. It is expected that Chileans older than 60 years old will increase from the current 15.7% of population to 32.9% by 2050, while people older than 80 years old will reach 10.3% [5].

In 1998, the Chilean Ministry of Health established a program for older adults designed to: (i) maintain the health and activity levels in later life, (ii) reduce the acute morbidity and functional decline, (iii) decrease the health inequalities and (iv) promote an adequate elderly nutrition through a complementary feeding program for older people (called PACAM) [6].

Since 1999, the PACAM has delivered foods to Chilean older adults who are beneficiaries of the National System of Health Services [7,8]. The primary food of the PACAM was a powdered soup (*Crema Años Dorados*). It is currently made with legumes and cereal flours and fortified with vitamins and minerals [9]. Later, the Department of Nutrition of the Faculty of Medicine from the University of Chile explored new complementary feeding options, incorporating until today a powdered dairy drink for older adults (*Bebida láctea Años Dorados*). This food is made from milk and cereals, fortified with minerals and vitamins, and flavoured with vanilla.

Today, the distribution of the PACAM foods through the Chilean territory is successful, reaching a delivery close to the 70% of the older population. However, the real effect of the intake of these foods on the nutritional status and micronutrients serum levels of the Chilean elderly is not fully elucidated. Only a few studies have evaluated the acceptability, tolerance, and consumption of the PACAM foods, and their real contribution on the daily feeding of their beneficiaries [6,9,10]. Regrettably, it was found that the PACAM foods did not contribute to maintaining an adequate serum level of some critical micronutrients such as vitamin B_12_, probably due to their insufficient absorption as consequence of the gastric atrophy of elderly [6].

Biologically, aging process is understood as the time related to decline of physiological functions, leading to changes in functional performance. Atrophic gastritis is one of the common changes in gastrointestinal physiology with advancing age, with an overall prevalence of 20% in elderly persons. Therefore, it could affect the bioavailability of specific nutrients, such as minerals and vitamins whose absorption is pH dependent [10]. Hence, all these physiological changes cause the deterioration of the nutritional status in elderly.

In Chile, one of the main challenges of nutritional intervention programs is to positively impact the nutritional status of the target population. The adequate design of policies and programs for the elderly should encourage a healthy aging despite increasing longevity. For that reason, the PACAM must satisfy the real nutritional needs of Chilean older population. Hence, the aim of the present work was to determine whether consumption of food distributed by the PACAM has a positive effect on the nutritional status, micronutrients intake and serum levels in older adults from the Metropolitan Region, Santiago de Chile.

## 2. Materials and Methods

### 2.1. Setting and Sampling

The testing of older adults was conducted in 2018 based on the 209 food distribution centres for elderlies financed by Chilean Government in the Metropolitan Region. The sample size was calculated based on the Chilean vitamin D deficit with a standard margin of error of 5 percentage points and a 95% confidence level, according to the size of the target population estimated by the Instituto Nacional de Estadísticas of Chile (INE, Santiago, Chile) for 2017 [4]. It was randomized according to the socioeconomic status of Chilean population.

### 2.2. Nutritional Status of the Elderly

Nutritional status was determined using the body mass index criteria (BMI). The BMI was calculated measuring the weight and the height according to the Chilean Health Ministry (MINSAL, Santiago, Chile) policy [11]. Weight and height were measured in barefoot volunteers in light clothing, using a balance (SECA 803, Hamburg, Germany, precision 0.1 kg) and an attached stadiometer (SECA 213, Hamburg, Germany, 0.1 cm). The BMI was classified according to the nutritional status in the following categories: ≤23 (underweight); >23.1 a 27.9 (normal); 28 a 31.9 (overweight) and ≥32 (obese) [11].

### 2.3. Dietary and Nutrients Intake of the Elderly

Dietary intake data were obtained using three 24 h food recalls (24-HR) performed on two nonconsecutive days within one week, considering weekend days proportionally to weekdays. Each recall was administered by trained interviewers, provided with standardized neutral probing questions according to the Multiple Pass Method (MPM) [12] and accompanied with the graphic portion’s booklet developed by the Universidad de Chile and MINSAL, to improve precision of the obtained information [13].

To standardize the collect measurements, the interviewers were trained by certified nutritionists who simultaneously worked as supervisors of the fieldwork. The collection measurements obtained in the 24-HR were converted to grams (g) and milliliters (mL) [14].

### 2.4. Serum Micronutrients Status of the Elderly

For the nutritional-metabolic characterization of the older adults enrolled to the PACAM, a baseline was established to define a specific micronutrients profile in older adults at serum level. For this purpose, blood samples were collected into vacutainer tubes without additives (Greiner Bio-One International AG, VACUETTE, Kremsmünster, Austria), and serum determinations of calcium and zinc were performed through TRXF methodology in the Laboratory of the Institute of Nutrition and Food Technology (INTA, Dhaka, Bangladesh). The determination of serum levels of vitamins A, B_12_ and 25-hydroxyvitamin D [25(OH)D] was performed through ECL method (electrochemiluminescence immunoassay) and the calibration curve was carried out by using commercial standards.

### 2.5. Statistical Methods

The normality of the continuous response variables (BMI, intake of micronutrients and serum micronutrient levels) of the elderly group was checked by Kolmogorov–Smirnov test (*n* ≥ 50) through SPSS 25.0 software (IBM, Corp., Armonk, NY, USA). Since all these variables were not normally distributed (nonparametric), comparisons between groups were made using the 2 independent samples Mann–Whitney test at *p* < 0.05.

The micronutrient intake and serum micronutrient levels by gender, age and PACAM consumption groups were presented as the median and in 25th–95th interquartile ranges. Spearman correlation analysis was done to assess the correlation between BMI with and serum 25-hydroxyvitamin D levels.

### 2.6. Ethical Considerations

All subjects gave their informed consent for inclusion before they participated in the study. The study was conducted in accordance with the Declaration of Helsinki, and the protocol was approved by the Ethics Committee of the Institute of Food Nutrition and Technology (INTA) of the University of Chile (Project identification code 20/2017).

## 3. Results

### 3.1. Sample Characteristics and Nutritional Status

The sample was comprised of 182 older adults (79% of women and 21% of men) from the Metropolitan Region, Santiago de Chile aged between 60 and 80 years. The 95% of the older adults were living with their relatives, and most of them were married (29%) or widowed (40%); while the 8 and 22% were divorced and single, respectively. On the other hand, an 80% of the older adults who studied had a complete basic education or a higher one, and 39% of them had technical and university studies.

According to the cut-off points established for the BMI in older adults, 13%, 46%, 28% and 13% of the sample were underweight; normal weight; overweight; and obese, respectively. Women presented 11% of underweight, 45% of normal weight and 44% of were overweight and obese, while men—18%, 50% and 32%, respectively.

### 3.2. Micronutrient Intake

The median micronutrient intakes of older adults (vitamin A: 381.67 µg/day, vitamin B_12_: 1.95 µg/day, vitamin D: 3.62 µg/day, calcium: 548.94 mg/day and zinc: 5.93 mg/day) were estimated based on the food consumption information recollected from the applied survey tests (Table 1). Only the 95th percentile of micronutrient intake were higher than their respective Recommended Daily Dose, while the 50th percentile (median values) were found to be below the Recommended Daily Dose for vitamin A (45%), vitamin B_12_ (19%), vitamin D (75%), calcium (46%) and zinc (26%) (Table 1).

There were no significant differences in micronutrient intake according to gender, with the exception of zinc that was 25% significantly higher (*p* < 0.05) in men (Table 2). On the other hand, the group of elderly people over 75 years old presented significantly higher (*p* < 0.05) intakes of vitamin A (27%), calcium (18%) and zinc (13%) intakes than 65–75 years old group (Table 3).

Regarding to the effect of the consumption of foods provided by the PACAM on micronutrient intake (Table 4), vitamin D and zinc intakes were significantly higher (*p* < 0.05) in those older adults who consumed these foods (47% and 22%, respectively). However, it is important to point out that even with the consumption of foods from the PACAM, many of the older adults evaluated in this study did not reach the micronutrient intakes Recommended Daily Dose. For all the groups, the 25th, 50th, 75th, and 95th percentiles of vitamin D intake were found to be below the recommended intake.

### 3.3. Serum Levels and Deficit of Micronutrients

Beyond the knowledge on the micronutrient intakes of the older adults, it is very important to determine their micronutrient serum levels to explore some possible micronutrient deficiencies. The median values of serum vitamin A, vitamin B_12_, vitamin D, calcium and zinc evaluated in the sample of elderly were 53 µg/dL, 301 pg/mL, 20.23 ng/mL, 8.74 mg/dL and 99.96 µg/dL, respectively (Table 1). The 25th percentiles of serum vitamin B_12_, 25-hydroxyvitamin D and calcium were lower than the normal values, while the 50th and 75th percentiles serum 25-hydroxyvitamin D were a 33% and 17% lower than the normal minimum reported (Table 1).

Regarding the effect of the gender on the micronutrient’s serum levels, women reached higher serum levels of vitamin B_12_ (5%) and calcium (3%) than men (Table 2). While men showed a higher serum concentration in vitamin A (11%), 25-hydroxyvitamin D (4%) and (0.6%) zinc (Table 2). However, no significant differences (*p* < 0.05) were observed between both gender groups (Table 2). In contrast, slight significant differences were observed between both evaluated age groups (Table 3). Specifically, for serum levels of vitamin B_12_ (1%), 25-hydroxyvitamin D (6%) and calcium (0.1%) which were significantly higher (*p* < 0.05) in the elderly over 75 years old (Table 3). On the other hand, the vitamin A and zinc serum levels were higher (3 and 4%, respectively) in the 60–75 years old group, however, only for serum zinc this increase was significant (*p* < 0.05).

In addition, regarding to the effect of PACAM foods consumption on the micronutrient serum levels; only the serum concentration of zinc was significantly higher (*p* < 0.05) in the non-consumers group (5%) (Table 4).

**Figure 1:**
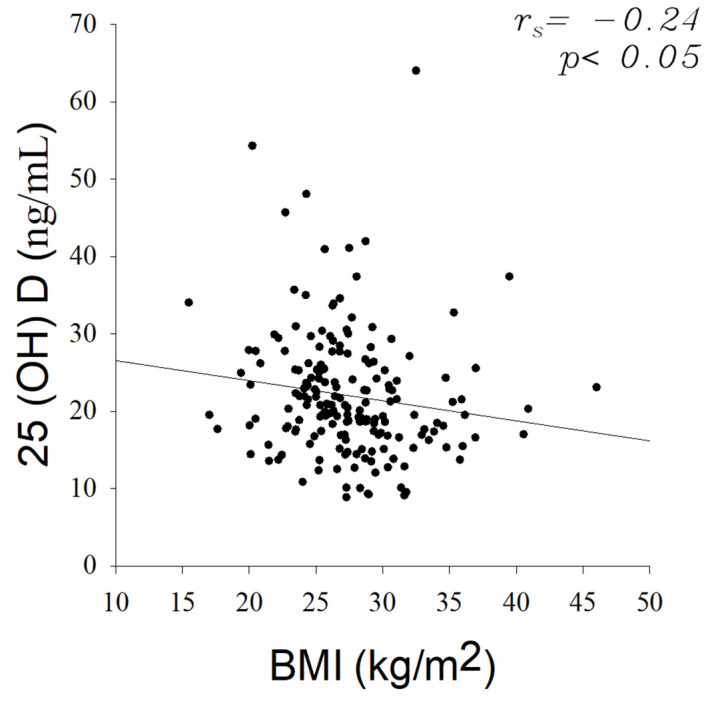
Scatterplot of body mass index (BMI) vs. plasma 25-hydroxyvitamin D. Spearman´s correlation coefficient is presented in the figure at *p* < 0.05.

Low serum micronutrient levels were reflected in severe serum deficits of 25-hydroxyvitamin D (88%), calcium (33%) and vitamin B_12_ (34%). Contrary, vitamin A and zinc serum presented low and null deficits, respectively. In this regard, the influence of the nutritional status, gender, age and foods PACAM consumption on the distribution of serum micronutrient deficiencies in elderly was analysed (Table 5). Older adults with normal nutritional status showed the highest percentages values for serum 25-hydroxyvitamin D (45%), vitamin B_12_ (43%), calcium (38%) and zinc (27%) deficiencies. Followed by overweight and obese (Table 5). In relation to this, BMI showed a slight significant inverse correlation (*p* < 0.05; rs = −0.24) with serum levels of 25-hydroxyvitamin D (Figure 1).

Regarding to gender, the highest percentages of serum micronutrient deficiencies were mainly detected in females (Table 5), showing deficit values of 79%, 75%, 73% and 73% for serum 25-hydroxyvitamin D, vitamin B_12_, calcium and zinc, respectively. On the other hand, older adults group aged between 60–75 years old exhibited the highest percentages of serum deficiencies for 25- hydroxyvitamin D (53%), vitamin B_12_ (61%) and zinc (55%) except for the calcium, that was equal in both age groups (50%). Contrary to micronutrient intake, all serum micronutrient deficiencies were majority detected in the consumers of PACAM foods group (Table 5), showing percentages values of 63% (25-hydroxyvitamin D), 59% (vitamin B_12_), 70% (calcium) and 64% (zinc) (Table 5).

## 4. Discussion

### 4.1. Nutritional Status

The screening of nutritional status in the elderly is crucial for the early detection of malnutrition and maintaining a good health and quality of life [18]. In this sense, simple anthropometric indices such as the BMI are essential tools for evaluating malnutrition, functional decline and chronic health conditions in geriatric assessment [19,20,21]. According to the BMI criteria, 46% of the older adults presented normal weight and 41% were overweight and obese. These values were different from the latest data reported by the National Health Survey [22] and other investigations carried out in Chile a few years ago [23,24], in which overweight and obese presented higher percentages of occurrence. It can be explained because these studies used the cut-off values indicated for adults. In our study the nutritional status of older adults was determined using the specific cut-off values proposed for this age group [11].

### 4.2. Micronutrient Intake

The dietary pattern in older adults is affected by ethnical, economics, cultural and social factors. The aging process involves important changes at the physiological level, such as: (i) sensory alterations (decreased of olfactory function), (ii) oral health deterioration (loss of teeth), (iii) reduced saliva production and (iv) problems of swallowing. All these dysfunctions significantly decrease the enjoyment and therefore the consumption of foods generating an insufficient intake of micronutrient in older adults [25,26,27]. Suboptimal micronutrient intake is reported to be common in older adults between 65–70 years old. It is associated with the “anorexia of aging”, defined as the loss of appetite and/or decreased food intake in late life [28].

Micronutrient intake, particularly of zinc was lower in elderly women group because of gender-specific differences in dietary habits. It was found that men consumed more food sources of zinc than women such as beef, chicken, and animal protein (Supplementary Material Table S1).

Our results agreed with other previous Chilean studies which reported low intakes of zinc in older adults aged over 60 years [23,29]. Low micronutrient intakes were also found in elderly studies from South Africa [30]), Chinese [31] and United Kingdom [32,33]. These similarities could be a consequence of the globalization and urbanization on the food supply, resulting in changes of dietary patterns and lifestyle behaviours during aging throughout the world.

Several dietary patterns have been associated with significant health benefits in the older population, especially those characterized by a high consumption of fruits and vegetables and low consumption of meat and processed foods [34]. In this study, the dietary pattern of elderly was characterized by: (i) a high consumption of carbohydrate-rich foods such as bread, rice, pasta, and potatoes; (ii) a moderate consumption of fruits and vegetables, and (iii) a very low consumption of dairy products (Table S1), which is in accordance with the previously reported information for Chilean older adults [35,36].

Only 63% of the older adults evaluated in this study consumed the PACAM foods, however they did not reach the recommended daily serving (50 g/day) of the *Años Dorados* dairy drink [37]. Interestingly, although the *Años Dorados* legume soup has been the worst sensory evaluated PACAM food, its consume was higher compared to dairy drink; reaching the 54 g/day and 74 g/day in female and male subjects, respectively (Table S1).

Most of the older adults did not consume these foods because: (i) they did not take it from the health centre, (ii) they did not like the taste or found it boring, (iii) they found difficulties diluting the dairy beverage, (iv) gastric discomfort and (v) lack of information (about recall and benefits).

### 4.3. Serum Levels and Deficit of Micronutrients

Vitamin D and calcium are essential micronutrient s whose absorption is strongly related. During aging there is insufficiency of both micronutrients, which combined with the age-related hormonal decline, negatively affect bone health in older adults [38,39]. Similarly, vitamin B_12_ deficiency is highly prevalent in the older adults leading to anaemia, neuropathy and neuropsychiatric disorders and nonspecific tiredness in older people [40,41].

This study found a high deficit of 25-hydroxyvitamin D, calcium and vitamin B_12_ in Chilean older adults. Previous studies in Chilean older adults reported similar serum micronutrient deficiencies [6,42]. These results could be associated with an insufficient intake from food sources and other physiological factors typical of aging, such as intrinsic factor deficiency, hypochlorhydria or food-bound malabsorption [43].

Serum micronutrient deficiencies are quite common in older adults regardless of their nutritional status. In this study, a weak significant negative relationship between the 25-hydroxyvitamin D serum levels and the BMI was found. Some researchers have found and inverse relationship between 25-hydroxyvitamin D levels and nutritional status through correlations with BMI [44,45,46,47]. Gonçalves et al. (2020) also found a direct association between hypovitaminosis D and obesity in elderly. However, although several studies confirmed that there is an inverse association between BMI/body fat content and serum 25-hydroxyvitamin D, the evidence is still not enough to confirm causality or collinearity [48]. Furthermore, both genders were deficient in serum 25-hydroxyvitamin D, vitamin B_12_ and calcium, but deficiencies were more pronounced in women, probably due to their dietary patterns.

Along with the above, during aging there is an inadequate intestinal absorption of micronutrients combined with a hormonal decline [49]. Vitamin D metabolism involves the hydroxylation to 25 hydroxyvitamin D (25OHD), and then it is metabolized to the hormonal active form 1,25-dihydroxyvitamin D (1,25(OH)_2_D). Thus, the form [1,25(OH)_2_D] is reduced which is the major controlling hormone of intestinal calcium absorption [50]. It is necessary to understand that calcium metabolism collaborates with other nutrients to create the bone matrix. Thus, calcium comprises a complex interactive dependency on the actions of phosphorus, vitamin D and proteins [51,52].

It is important to point out that the consumption of the PACAM foods did not improve the micronutrient status of older adults. Similar results were previously reported when the effectiveness of the PACAM foods on the serum status of vitamin B_12_ in Chilean older adults was evaluated [6]. In older adults, serum vitamin B_12_ deficiency can be caused by (i) a poor consumption of animal foods and/or (ii) physiological alteration of the gastrointestinal mucosa, which leads to an absorption and transport disorders. Then, it is necessary to introduce it through supplements or fortified foods [53].

Typical changes of the aging process such as reduced appetite, lack of acid gastric and enzyme secretions have important consequences on the digestive absorption of some vitamins and trace elements [26,54], as well as the coexistence of these changes together with the use of drugs associated to certain pathologies [27]. Another important remark is that minerals bioavailability from plants and many staple foods such cereals and legumes can be affected by their contents of oxalate and phytate, which are inhibitors of zinc, iron and specially calcium absorption content [55,56]. Besides, it was found that phytates and tannins, as well as fibres and saponins, can negatively influence the bioavailability of fat-soluble vitamins such as vitamin D, possibly due to modulation of lipolysis during digestion [57]. Accordingly, vitamins and minerals supplementation through food matrices such as the powdered soup *Años Dorados* must consider a decreased absorption of mineral due to the chelating effect of the food ingredients.

## 5. Conclusions

Chilean older adults from the Metropolitan Region, Santiago, were characterized by a similar proportion of normal weight and overweight/obese subjects, being underweight the nutritional status with lowest occurrence. Although PACAM foods consumption was lower than their recommended servings, it significantly increased the micronutrient intake in older adults. In contrast, important serum deficits of vitamin D, vitamin B_12_ and calcium were detected both for consumers and no consumers of PACAM foods, reflecting that the intake of these products does not have any positive effect on the micronutrient serum levels of elderly. This study provides for the first-time valuable information on the real nutritional benefits of the PACAM and the goals and challenges for improving the nutritional status of Chilean older adults. Public health programs must receive constant feedback from nutritional intervention studies to rationally design foods that really promote successful aging.

## Figures and Tables

**Table 1:** BMI, micronutrient intake and serum micronutrient levels of an elderly group from Metropolitan Region, Santiago, Chile.

Evaluated Parameters		Elderly Group			
		*n* = 182			
		25th Percentile	Median	75th Percentile	95th Percentile
BMI (kg/m^2^)		24.85	27.22	29.61	35.92
Micronutrient intake	Recommended Daily Dose [15,16]				
Vitamin A (µg/day)	700 (women) and 900 (men)	246.06	381.67	596.45	1104.46
Vitamin B_12_ (µg/day)	2.40	1.29	1.95	2.89	4.81
Vitamin D_3_ (µg/day)	15	0.94	3.62	6.60	11.19
Calcium (mg/day)	1200	359.67	548.94	762.66	1348.73
Zinc (mg/day)	8 (women) and 11 (men)	4.33	5.93	7.91	10.84
Plasma micronutrient level	Normal values [17]				
Vitamin A (µg/dL)	20–100	47.00	53.00	67.00	85.30
Vitamin B_12_ (pg/mL)	279–996	255.50	301.00	383.00	627.20
[25(OH)D] (ng/mL)	30–100	16.88	20.23	25.37	37.34
Calcium (mg/dL)	8.5–10.5	8.31	8.74	9.23	10.35
Zinc (µg/dL)	75–120	89.39	99.96	113.40	158.69

**Table 2:** Influence of the gender on the BMI, micronutrient intake and serum micronutrient s levels of elderly from Metropolitan Region, Santiago, Chile.

Evaluated Parameters		Gender Group								*p*-Value
		Males				Females				
		*n* = 38				*n* = 144				
		25th Percentile	Median	75th Percentile	95th Percentile	25th Percentile	Median	75th Percentile	95th Percentile	
BMI (kg/m^2^)		24.03	26.85	28.99	35.08	24.94	27.27	30.13	35.94	0.26
Micronutrient intake	Recommended Daily Dose [15,16]	25th percentile	Median	75th percentile	95th percentile	25th percentile	Median	75th percentile	95th percentile	*p*-value
Vitamin A (µg/day)	700 (women) and 900 (men)	183.00	367.69	509.06	893.05	251.11	381.67	613.92	1229.34	0.52
Vitamin B_12_ (µg/day)	2.40	1.49	1.96	2.89	3.90	1.20	1.92	2.90	5.13	0.62
Vitamin D_3_ (µg/day)	15	0.78	3.48	6.79	9.66	0.91	3.47	6.33	11.43	0.52
Calcium (mg/day)	1200	322.08	477.15	723.32	1207.11	369.46	542.29	741.80	1443.72	1.00
Zinc (mg/day)	8 (women) and 11 (men)	5.03	7.19	9.49	10.97	4.08	5.76	7.34	11.08	0.02 *
Serum micronutrient level	Normal values [17]	25th percentile	Median	75th percentile	95th percentile	25th percentile	Median	75th percentile	95th percentile	*p*-value
VitaminA (µg/dL)	20–100	46.00	58.00	71.00	102.80	47.00	52.00	66.00	84.10	0.11
Vitamin B_12_ (pg/mL)	279–996	243.00	290.00	350.00	906.80	257.50	304.50	390.25	623.70	0.21
[25(OH)D] (ng/mL)	30–100	17.60	20.72	25.32	34.64	16.87	19.93	25.43	40.89	0.45
Calcium (mg/dL)	8.5–10.5	8.23	8.53	9.11	14.25	8.34	8.79	9.24	10.34	0.22
Zinc (µg/dL)	75–120	88.51	100.29	116.07	168.05	89.58	99.95	113.21	167.66	0.85

* Superscript symbols indicate significant differences (*p* < 0.05, Mann–Whitney Test).

**Table 3:** Influence of the age group on the BMI, micronutrient intake and serum micronutrient levels of elderly from Metropolitan Region, Santiago, Chile.

Evaluate Parameters		Age Group								*p*-Value
		60–75 Years				>75 Years				
		*n* = 99				*n* = 83				
		25th Percentile	Median	75th Percentile	95th Percentile	25th Percentile	Median	75th Percentile	95th Percentile	
BMI (kg/m^2^)		25.27	27.33	30.52	36.1651	24.32	26.81	28.76	35.4204	0.05
Micronutrient intake	Recommended Daily Dose [15,16]	25th percentile	Median	75th percentile	95th percentile	25th percentile	Median	75th percentile	95th percentile	*p*-value
Vitamin A (µg/day)	700 (women) and 900 (men)	210.71	354.56	494.97	972.18	260.89	450.01	673.31	1676.77	0.01 *
Vitamin B_12_ (µg/day)	2.40	1.13	1.88	2.69	5.19	1.45	2.06	3.04	4.85	0.16
Vitamin D_3_ (µg/day)	15	0.64	3.12	6.01	9.95	1.39	3.89	7.32	13.08	0.05
Calcium (mg/day)	1200	303.70	497.18	699.84	1052.58	411.32	588.36	857.31	1570.49	0.04 *
Zinc (mg/day)	8 (women) and 11 (men)	3.88	5.52	7.23	9.30	4.91	6.25	8.85	11.54	0.02 *
Serum micronutrient level	Normal values [17]	25th percentile	Median	75th percentile	95th percentile	25th percentile	Median	75th percentile	95th percentile	*p*-value
Vitamin A (µg/dL)	20–100	46.25	53.50	68.00	90.60	47.00	52.00	65.50	80.40	0.47
Vitamin B_12_ (pg/mL)	279–996	252.00	300.50	376.50	641.60	270.50	304.00	393.00	649.80	0.47
[25(OH)D] (ng/mL)	30–100	16.77	19.54	24.24	41.17	17.34	20.72	27.14	35.00	0.69
Calcium (mg/dL)	8.5–10.5	8.31	8.73	9.26	10.25	8.32	8.74	9.19	10.66	0.83
Zinc (µg/dL)	75–120	90.47	101.84	119.48	169.16	88.13	97.76	109.14	150.59	0.01 *

* Superscript symbols indicate significant differences (*p* < 0.05, Mann–Whitney Test).

**Table 4:** Influence of the PACAM foods consumption on the BMI, micronutrient intake and serum micronutrient levels of elderly from Metropolitan Region, Santiago, Chile.

Evaluated Parameters		Food PACAM Consumption Group								*p*-Value
		Consumes				Does not Consume				
		*n* = 115				*n* = 67				
		25th Percentile	Median	75th Percentile	95th Percentile	25th Percentile	Median	75th Percentile	95th Percentile	
BMI (kg/m^2^)		25.04	27.22	29.72	35.94	24.35	27.06	29.62	35.54	0.38
Micronutrient intake	Recommended Daily Dose [15,16]	25th percentile	Median	75th percentile	95th percentile	25th percentile	Median	75th percentile	95th percentile	*p*-value
Vitamin A (µg/day)	700 (women) and 900 (men)	241.90	381.32	612.20	1388.47	250.86	373.69	497.35	926.21	0.27
Vitamin B_12_ (µg/day)	2.40	1.30	1.99	2.84	4.60	1.17	1.90	3.06	5.40	0.71
Vitamin D_3_ (µg/day)	15	1.31	3.93	6.69	12.38	0.63	2.68	5.15	10.21	0.03 *
Calcium (mg/day)	1200	392.40	555.72	762.73	1458.51	308.89	475.66	700.17	1015.18	0.07
Zinc (mg/day)	8 (women) and 11 (men)	4.92	6.28	8.52	11.14	3.89	5.13	6.58	9.95	0.00 *
Serum micronutrient level	Normal values [17]	25th percentile	Median	75th percentile	95th percentile	25th percentile	Median	75th percentile	95th percentile	*p*-value
Vitamin A (µg/dL)	20–100	47.00	55.00	67.00	86.40	46.00	51.50	67.25	85.65	0.23
Vitamin B_12_ (pg/mL)	279–996	263.00	301.00	375.00	587.20	242.75	306.50	387.00	758.70	0.77
[25(OH)D] (ng/mL)	30–100	16.89	20.39	24.24	37.34	16.85	19.45	26.13	39.04	0.61
Calcium (mg/dL)	8.5–10.5	8.31	8.69	9.11	10.15	8.32	8.79	9.34	11.70	0.41
Zinc (µg/dL)	75–120	87.96	98.85	109.84	142.89	93.27	103.47	119.63	238.52	0.01 *

* Superscript symbols indicate significant differences (*p* < 0.05, Mann–Whitney Test).

**Table 5:** Influence of the nutritional status, gender, age and PACAM foods consumption on the micronutrient serum deficiencies of Chilean older adults.

GROUPS		[25(OH)D] (<30 ng/mL)	Vitamin B_12_ (<279 ng/mL)	Calcium (<8.5 mg/dL)	Zinc (<75 µg/L)
Nutritional status	Underweight	12.42% (*n* = 20)	14.75% (*n* = 9)	12.12% (*n* = 8)	36.36% (*n* = 4)
	Normal	44.72% (*n* = 72)	42.62% (*n* = 26)	37.88% (*n* = 25)	27.27% (*n* = 3)
	Overweight	29.81% (*n* = 48)	27.87% (*n* = 17)	33.33% (*n* = 22)	27.27% (*n* = 3)
	Obese	13.04% (*n* = 21)	14.75% (*n* = 9)	16.67% (*n* = 11)	9.09% (*n* = 1)
Gender	Male	21.12% (*n* = 34)	24.59% (*n* = 15)	27.27% (*n* = 18)	27.27% (*n* = 3)
	Female	78.88% (*n* = 127)	75.41% (*n* = 46)	72.73% (*n* = 48)	72.73% (*n* = 8)
Age group	60–75	53.42% (*n* = 86)	60.66% (*n* = 37)	50% (*n* = 33)	54.55% (*n* = 6)
	>75	46.58% (*n* = 75)	39.34% (*n* = 24)	50% (*n* = 33)	45.45% (*n* = 5)
PACAM consumption	Consumes	63.35% (*n* = 102)	59.02% (*n* = 36)	69.70% (*n* = 46)	63.64% (*n* = 7)
	Does not consume	36.65% (*n* = 59)	40.98% (*n* = 25)	30.30% (*n* = 20)	36.36% (*n* = 4)

## Data Availability

All data analyzed in this study are available upon request to the corresponding author.

## Supplementary Materials

The following are available online at https://www.mdpi.com/article/10.3390/nu14010003/s1, Table S1: Average food intake values in a group of older adults from Metropolitan Region, Santiago, Chile.
