# Supplementary material for: Nutritional Status and Serum Levels of Micronutrients in an Elderly Group Who Participate in the Program for Complementary Food in Older People (PACAM) from the Metropolitan Region, Santiago de Chile
Source: Nutrients. 2021 Dec 21;14(1):3. doi: 10.3390/nu14010003 (PMC8746835; doi:10.3390/nu14010003)
Supplement: Supplementary file 1 [file nutrients-14-00003-s001.zip › nutrients-1511145-supplementary.pdf]

Table S1. Food intake of a group of older adults from Metropolitan Region, Santiago, Chile

Food group	Gender group							
	Males				Females			
	n = 38				n = 144			
	25th percentile	Median	75th percentile	95th percentile	25th percentile	Median	75th percentile	95th percentile
Milk drink *Años Dorados*	25.3	39.2	48.3	51.0	15.1	37.2	45.3	57.0
Legume soup *Años Dorados*	54.0	74.0	84.2	100.1	27.5	54.0	67.0	83.0
Bread	80.0	142.9	184.6	220.4	59.5	100.3	115.3	141.4
Rice. pasta and cereal products	19.9	32.5	55.0	77.7	13.2	25.0	50.0	77.2
Potato based products	77.8	143.9	165.2	174.0	60.5	114.9	165.0	189.3
Fruits	29.7	95.6	194.0	251.1	49.5	106.8	184.3	264.3
Vegetables	25.6	91.2	155.3	221.4	39.1	88.2	144.1	275.9
Beef	36.4	54.3	100.0	116.0	28.8	50	91.5	98.2
Chicken	42.7	59.0	98.0	118.7	42.7	49	64	118.7
Sausages	16.6	33	33	68.0	15.0	33	50	60.0
Low fat milk	0	43	119	359.5	143.0	134.3	155.0	160.6
Legumes	19.8	35	49.25	67.4	12.8	35.0	40.2	72.2
Sugar	2.0	9	13.0	29.6	1.9	7.3	11.1	14.7
Oil	3.5	5	7.5	13.1	2.7	5	6.6	11.3
